# Thymic stromal lymphopoietin as a therapeutic target in patients with chronic rhinosinusitis and nasal polyps

**DOI:** 10.1093/cei/uxaf041

**Published:** 2025-09-05

**Authors:** Anju T Peters, Joseph K Han, Enrico Heffler, Freyja McClenahan, Scott Caveney, Tham T Le, Ayman Megally, Joseph D Spahn, Andrew Foster, Joseph D Sherrill

**Affiliations:** Division of Allergy and Immunology, Feinberg School of Medicine, Northwestern University, Chicago, IL, USA; Department of Otolaryngology – Head & Neck Surgery, Eastern Virginia Medical School, Norfolk, VA, USA; Personalized Medicine Asthma and Allergy Unit, IRCCS Humanitas Research Hospital, Milan, Italy; Department of Biomedical Sciences, Humanitas University, Milan, Italy; PharmaGenesis London, London, UK; Global Development, Inflammation, R&D, Amgen, Thousand Oaks, CA, USA; BioPharmaceuticals Medical, AstraZeneca, Gaithersburg, MD, USA; Late-Stage Development, Respiratory and Immunology, BioPharmaceuticals R&D, AstraZeneca, Gaithersburg, MD, USA; Respiratory and Immunology, BioPharmaceuticals Medical, AstraZeneca, Wilmington, DE, USA; Respiratory and Immunology, BioPharmaceuticals Medical, AstraZeneca, Gaithersburg, MD, USA; Translational Science and Experimental Medicine, Research and Early Development, Respiratory and Immunology, BioPharmaceuticals R&D, AstraZeneca, Gaithersburg, MD, USA

**Keywords:** cytokines, eosinophils, mucosa, inflammation

## Abstract

Chronic rhinosinusitis with nasal polyps (CRSwNP) is an inflammatory disorder of the sinonasal mucosa, predominantly characterized by epithelial dysfunction and chronic heterogeneous mucosal inflammation. CRSwNP and asthma are common comorbidities with overlapping pathophysiology, epithelial impairment, and activation of downstream type 2 inflammation. Thymic stromal lymphopoietin (TSLP) is an epithelial cytokine that sits at the top of the immunological cascade and initiates and amplifies type 2-dependent and -independent inflammatory responses. Although the role of TSLP in asthma has been well described, the role of TSLP in CRSwNP has yet to be comprehensively outlined. This review examines the evidence for TSLP as a key factor in CRSwNP pathogenesis. We explore what is known about TSLP expression patterns within the sinonasal mucosa, finding that TSLP expression is increased in patients with CRSwNP compared with healthy patients, and in eosinophilic- versus non-eosinophilic CRSwNP. We discuss the impact of environmental triggers and genetic factors on TSLP expression and activity, as well as other upstream regulators of TSLP signaling. We then consider the known mechanisms and effects of TSLP signaling on the recruitment and activation of various immune and structural cell types in CRSwNP. Finally, we consider the available evidence on the therapeutic potential of targeting TSLP signaling for the treatment of CRSwNP and discuss ongoing trials of promising therapeutic candidates.

## Teaser

Thymic stromal lymphopoietin (TSLP) is an epithelial cytokine that initiates and amplifies type 2-dependent and -independent inflammatory responses. This review explores the roles of TSLP signaling in the pathogenesis of chronic rhinosinusitis with nasal polyps and considers the therapeutic potential of targeting TSLP signaling for the treatment of this condition.

## Introduction

Chronic rhinosinusitis (CRS) is a group of inflammatory disorders of the sinonasal mucosa. Although CRS is mechanistically heterogeneous, having various endotypes and phenotypes, it generally involves immune and epithelial barrier dysfunction combined with genetic, microbial, and environmental factors that drive pathologic inflammatory processes [1]. Symptoms of CRS can include nasal congestion, rhinorrhoea or postnasal drainage, facial pressure or pain, and/or anosmia lasting for longer than 12 weeks. Secondary consequences of CRS include sleep impairment, anxiety, and depression [2, 3]. CRS affects 5%–12% of the general population and both symptoms and secondary consequences can have a significant impact on health-related quality of life [3–5].

Approximately 20%–30% of patients in Western countries (including the USA, European countries, and Australia) with CRS have nasal polyps (NPs), inflammatory epithelial extrusions originating from the ethmoid sinus epithelium and projecting into the nasal airway [4, 6, 7]. Patients with CRS and NPs (CRSwNP) have more severe sinonasal symptoms than those without NPs [7]. Common symptoms reported among patients with CRSwNP include nasal congestion, anosmia, sneezing, and headache, with results varying widely between studies and across geographical populations [8].

### Endotypes–phenotypes of CRS and associated clinical characteristics

Historically, CRS has been classified based on the presence or absence of NPs (CRSwNP and CRSsNP, respectively). However, the patterns of inflammation within each phenotype are heterogeneous, and multiple overlapping inflammatory pathways may be observed. Type 2 (T2) inflammation, featuring eosinophilia and elevated levels of interleukin (IL)-5 and IL-13, is a common component of CRSwNP and is present in approximately 69%–90% of patients from Western countries [9–11]. Many patients with CRSwNP, though, exhibit a mix of T1, T2, and T3 endotypes, with the proportions of each endotype varying by geography [12–14]. In Asian countries, CRSwNP has generally been associated with non-eosinophilic presentation with a mix of T1 and T3 signatures [9, 15]. However, in a 2016 study of T helper (Th) cytokine profiles, 57% of patients from Beijing, China, showed evidence of a mixture of two or more inflammatory endotypes, including T2, and more than 50% of patients from Tochigi, Japan, had eosinophilic/T2 CRSwNP (eCRSwNP) [10]. Other patients with CRSwNP have no distinct inflammatory profile (termed Tun or ‘untypeable’) [10, 16]. Allergic rhinitis (AR) is a highly prevalent comorbidity, with approximately 51%–86% of CRSwNP patients being sensitized to at least one aeroallergen [10, 14, 16]. CRSsNP, meanwhile, was thought to be driven primarily by T1 inflammatory processes; however, recent studies have also implicated T2 inflammation in many CRSsNP patients [10, 17, 18].

The different inflammatory endotypes in both CRSwNP and CRSsNP are associated with distinct clinical characteristics. Anosmia, asthma comorbidity, allergic symptoms, and the presence of mucin are significantly associated with the T2 endotype, the T1 endotype is significantly more common in females than males and is associated with ear/facial pain and pressure, and the presence of pus is significantly associated with the T3 endotype [14, 16].

### The united airway

CRSwNP is significantly associated with asthma and AR, with approximately 24%–56% of Western patients and 7%–23% of patients in Asian countries with CRSwNP estimated to have comorbid asthma, and approximately 26%–76% and 17%, respectively, estimated to have comorbid AR. However, estimates of AR comorbidity may be confounded by significant symptom overlap between CRSwNP and AR [6, 8]. Meanwhile, approximately 20%–40% of patients with severe asthma worldwide have CRSwNP [19, 20].

Comorbid asthma among patients with CRSwNP tends to be of an adult-onset T2 endotype and is more likely to be severe and difficult to control than in patients with asthma alone [1]. Patients with CRSwNP and comorbid asthma also have more severe CRS, greater recurrence of NPs, and higher rates of corticosteroid dependence than those with asthma alone [1]. A subtype of CRSwNP and comorbid asthma is aspirin/NSAID-exacerbated respiratory disease (AERD), or Samter’s triad, which is characterized by hypersensitivity to cyclooxygenase-1 inhibitors leading to airway hyperreactivity. CRSwNP in patients with AERD typically features more severe tissue eosinophilia and T2 inflammation than in those without AERD [1].

**Figure 1.**
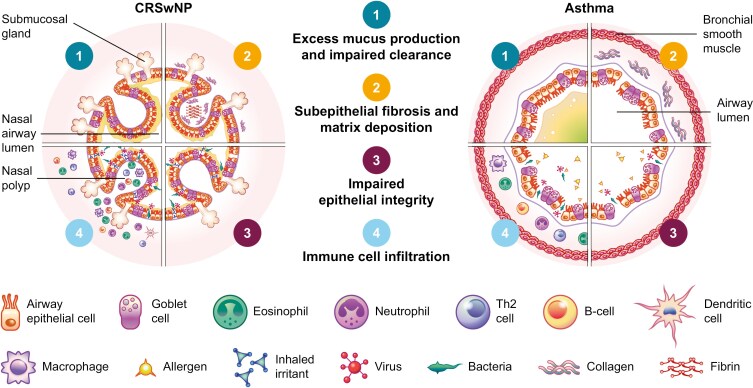
CRSwNP and asthma share similar pathophysiological features of airway remodeling and inflammation. Epithelial damage leads to epithelial cytokine release and immune cell infiltration, triggering downstream processes such as mucus overproduction, fibrosis, and remodeling of the underlying tissue. Chronic inflammation and tissue changes prevent effective epithelial repair, leading to a vicious cycle that drives the symptoms of airway disease. CRSwNP, chronic rhinosinusitis with nasal polyps; Th, T helper.

The shared anatomy of the sinonasal and bronchial epithelia contributes to the significant comorbidity of CRSwNP in the upper airways and asthma in the lower airways (**Figure 1**) [6, 21]. The pathophysiology of both CRSwNP and asthma involves the interaction between environmental stimuli, a pro-inflammatory state, and an impaired epithelial barrier [1, 22–24]. Like T2 (adult-onset) asthma, eCRSwNP features elevated levels of key T2 cytokines (including IL-4, IL-5, and IL-13) and chemokines (such as C-C motif ligand [CCL] 2, -11, -17, -18, -24, and -26) compared with healthy patients [1]. Elevated levels of dendritic cells (DCs), group 2 innate lymphoid cells (ILC2s), macrophages, and mast cells have also been found in NP tissue of patients with CRSwNP [1, 25] and bronchial tissue and sputum from those with asthma [26–29].

Many of the epithelial barrier abnormalities observed in the sinonasal epithelium in patients with CRSwNP are also seen in the bronchial epithelium of patients with asthma, including tight junction defects, basal cell dysplasia, cilia loss, impaired secretory cell function, subepithelial extracellular matrix deposition, and epithelial-to-mesenchymal transition (EMT) [30]. In CRSwNP, epithelial barrier defects are also a cause and a consequence of colonization by pathogens, such as *Staphylococcus aureus*, which can trigger innate and adaptive immune responses and exacerbate ongoing inflammation [1, 31]. When pathogens, proteases, and irritants breach the epithelial barrier, the epithelial cells respond by producing cytokines, such as thymic stromal lymphopoietin (TSLP), IL-25, and IL-33, of which TSLP is the most strongly induced [1, 30].

### Introduction to TSLP

TSLP is a key upstream initiator and amplifier of innate and adaptive immune responses [9, 32, 33]. In response to epithelial damage or insult, epithelial cells release TSLP (along with IL-25 and IL-33) to initiate and amplify downstream inflammation through T2 and T2-independent pathways [9, 32, 33]. TSLP stimulates the innate immune response by polarizing DCs via the upregulation of OX40 ligand (OX40L), driving the differentiation of naive T cells to Th2 cells, and stimulating the production of T2 cytokines from ILC2s and Th2 cells [32–34]. Additionally, TSLP can act directly on downstream effector/mediator cells, including eosinophils, mast cells, basophils, and macrophages, to further propagate and amplify inflammation through local T2 cytokine production [32, 33]. Furthermore, TSLP plays a role in airway remodeling processes via effects on structural cells including fibroblasts [32, 35]. Airway epithelial cells produce TSLP upon exposure to a wide variety of environmental insults (including viruses, allergens, bacteria, smoke, and pollution), as well as to pro-inflammatory cytokines (such as tumor necrosis factor [TNF]-α and IL-1β) [33].

In this review, we examine the role of TSLP in the pathobiology of CRSwNP and its potential as a therapeutic target in this disease. To inform the review, we conducted a literature search of the PubMed database for articles in English published before 7 February 2024 using search terms (TSLP [all fields] OR thymic stromal lymphopoietin [all fields] AND chronic rhinosinusitis [all fields] OR CRSwNP [all fields] OR nasal polyp* [all fields]), employing the ‘Humans’ species filter and excluding review articles. The results from this search were screened for relevance (i.e. whether they contained information about TSLP expression, TSLP regulation, TSLP effector cells, or physiological or clinical effects of TSLP in patients with CRSwNP) and were supplemented by further relevant articles known to the authors.

## Expression and activity of TSLP in the nasal epithelium of patients with CRS

Overall, studies have shown that TSLP messenger RNA (mRNA) and protein are elevated in patients with CRSwNP versus CRSsNP, particularly in the T2/eosinophilic endotype (summarized in Table 1). TSLP expression has primarily been localized to nasal epithelial cells (NECs) and basal stem cells but has also been reported in subepithelial fibroblasts and infiltrating inflammatory cells.

**Table 1.** TSLP mRNA and protein expression in CRSwNP studies

Reference	Patient population	CRS phenotype/endotype	Sample site	TSLP expression	Technique
Nagarkar et al. 2013 [36]	USA	CRSwNP vs CRSsNP vs healthy controls	NP tissue from CRSwNP, UT from CRSsNP, and control	TSLP and TSLPR mRNA increased in NP tissue from CRSwNP versus UT from CRSsNP and controls. Full-length protein decreased in NP tissue from CRSwNP versus CRSsNP and controls. Cleaved TSLP had greater biological activity than full-length TSLP	RT-PCR, ELISA, WB
Boita et al. 2015 [37]	Italy	CRSwNP vs CRSsNP vs healthy controls	NP tissue in CRSwNP, OMC in CRSsNP, and controls	TSLP and TSLPR mRNA increased in CRSwNP versus control. No difference in mRNA between CRSwNP and CRSsNP	RT-PCR
Liu et al. 2021 [38]	China	CRSwNP vs CRSsNP vs healthy controls	NP tissue from CRSwNP, UT from CRSsNP, and turbinates from controls. NECs from the nasal mucosa	mRNA increased in CRSwNP versus controls	qRT-PCR
Wang et al. 2023^a^ [39]	USA	CRSwNP vs CRSsNP vs healthy controls	NP tissue from CRSwNP, UT from CRSsNP and controls, and primary NECs from NPs	TSLP mRNA increased in NP tissue and NECs isolated from NP tissue from CRSwNP versus control tissue and healthy controls	qRT-PCR
Boita et al. 2011 [40]	Italy	CRSwNP vs CRSsNP vs healthy controls	NP tissue in CRSwNP, OMC mucosa in CRSsNP, and nasal mucosa in controls	TSLPR protein increased in NECs and inflammatory infiltrate in CRS vs controls, with no difference in CRSwNP vs CRSsNP	IHC
Workman et al. 2019 [41]	USA	CRSwNP vs healthy controls	NP tissue from CRSwNP and inferior turbinate tissue from controls	TSLP mRNA increased versus controls; protein unchanged	Matched tissue proteomic and transcriptomic array
Dogan et al. 2019 [42]	Turkey	CRSwNP vs healthy controls	NP tissue from CRSwNP or sinonasal tissue from controls	TSLP protein increased in CRSwNP versus controls	ELISA
Ogasawara et al. 2018 [43]	USA	CRSwNP	NP tissue and control ethmoid sinus tissue	Total TSLP mRNA was higher in NP tissue than in control tissue. mRNA for the long variant of TSLP was elevated in NP versus control ethmoid tissue	RT-PCR
Liu et al. 2011 [44]	China	CRSwNP vs CRSsNP	Nasal mucosal tissue	TSLP mRNA and protein increased in CRSwNP vs CRSsNP in the epithelial layer. TSLPR increased in DCs of NP nasal mucosa versus CRSsNP	qRT-PCR, WB, ELISA
Shiozawa et al. 2015 [45]	Japan	CRSwNP vs healthy controls	Primary NECs from NPs in CRSwNP or inferior concha (turbinate) membrane in controls	TSLP protein secretion increased in CRSwNP vs controls	Bio-Plex cytokine assay
Wang et al. 2023b [46]	USA	CRSwNP vs CRSsNP	Primary NECs	Basal cells expressing TSLP are increased in CRSwNP vs CRSsNP and express higher levels of TSLP	RNA-seq (single-cell and bulk)
Lam et al. 2013 [47]	Australia	CRSwNP vs CRSsNP vs healthy controls	Sinus mucosal samples (maxillary or ethmoidal)	No difference in mRNA between patients with CRSwNP, CRSsNP, and controls	qRT-PCR
Lam et al. 2015 [48]	Australia	CRSwNP vs CRSsNP vs healthy controls	Sinus mucosal samples (maxillary or ethmoidal)	No difference in mRNA between CRSwNP and CRSsNP or controls. mRNA decreased in CRSsNP vs control	qRT-PCR
Lee et al. 2017 [49]	Taiwan	Severe or non-severe asthma and CRSwNP	Middle turbinate tissue	TSLP mRNA and protein increased in severe asthma and CRS vs non-severe asthma and CRS	qRT-PCR, IHC
Türk et al. 2022 [50]	Turkey	Severe asthma with CRSwNP vs healthy controls	Serum	No difference in serum TSLP levels between CRSwNP and control	ELISA
Shi et al. 2014 [51]	China	eCRSwNP^a^ vs neCRSwNP vs healthy controls	NP tissue from CRSwNP, inferior turbinate mucosa from controls	mRNA increased in eCRSwNP vs neCRSwNP and controls. mRNA correlated with OX40L+ DCs in sinonasal mucosa	qRT-PCR
Kobayashi et al. 2020 [52]	Japan	eCRSwNP^b^ vs neCRSwNP	NP or uncinate process tissue from CRSwNP, NECs from inferior turbinate	mRNA increased in NPs compared with uncinate process tissues from the same patient	qRT-PCR
Liao et al. 2015 [53]	China	eCRSwNP^a^ vs neCRSwNP vs healthy controls	NP tissue from CRSwNP, ethmoid sinus mucosa from controls	TSLP/TSLPR mRNA and protein increased in eCRSwNP vs neCRSwNP and controls. In eCRSwNP, TSLP was expressed in mast cells and TSLPR was expressed in mast cells, DCs, macrophages, and CRTH2+ cells	qRT-PCR, ELISA, IHC, flow cytometry
Nagata et al. 2019 [54]	Japan	eCRSwNP^c^ vs neCRSwNP vs healthy controls	NP tissue from CRSwNP or middle meatus tissue from controls	TSLP protein increased in eCRSwNP versus control but not in eCRSwNP versus neCRSwNP	IHC
Shin et al. 2020 [55]	Korea	eCRSwNP^d^ vs neCRSwNP vs healthy controls	NP tissue from CRSwNP, uncinate tissue from controls	TSLP protein increased in eCRSwNP vs neCRSwNP and controls. TSLP protein increased in neCRSwNP vs controls	ELISA
Kouzaki et al. 2016 [56]	Japan	eCRSwNP^e^ vs neCRSwNP	Primary NECs from nasal tissue	TSLP mRNA increased in NECs from eCRSwNP vs neCRSwNP. TSLP protein was increased in NPs from eCRSwNP vs neCRSwNP	RT-PCR, ELISA, IHC
Ouyang et al. 2013 [57]	China	eCRS^f^ vs neCRS	Ethmoid sinus mucosa	TSLP and TSLPR mRNA and protein increased in eosinophils in eCRS vs neCRS. TSLP and TSLPR protein increased in atopic versus non-atopic patients. TSLP associated with NP scores	RT-PCR, IHC

^a^eCRSwNP was classified based on the proportion of eosinophils exceeding 10% of total infiltrating cells.

^b^eCRSwNP was characterized by ethmoid-predominant sinusitis with eosinophilic inflammation.

^c^eCRSwNP was classified based on the number of eosinophils in 6 (400×) fields being ≥70.

^d^eCRSwNP was classified based on the presence of ≥10 eosinophils per high-power field.

^e^eCRSwNP definition was not provided.

^f^eCRS was classified based on the presence of ‘numerous eosinophils’ and EG2 expression. neCRS was classified based on the presence of ‘greater numbers of neutrophils and monocytes’.

CRS, chronic rhinosinusitis; CRSwNP, CRS with NP; CRSsNP, CRS without NP; CRTH2, chemoattractant receptor-homologous molecule expressed on Th2 cells; DC, dendritic cell; eCRS, eosinophilic CRS; eCRSwNP, eosinophilic CRSwNP; ELISA, enzyme-linked immunosorbent assay; IHC, immunohistochemistry; NEC, nasal epithelial cell; neCRS, non-eosinophilic CRS; neCRSwNP, non-eosinophilic CRSwNP; NP, nasal polyp; OMC, ostiomeatal complex; RNA-seq, RNA sequencing; RT-PCR, reverse transcriptase polymerase chain reaction; qRT-PCR, quantitative RT-PCR; TSLP, thymic stromal lymphopoietin; TSLPR, TSLP receptor; UT, uncinate tissue; WB, western blot.

### TSLP mRNA

Almost all relevant studies identified for this review found that TSLP mRNA expression was increased in NP tissue of patients with CRSwNP compared with nasal mucosa tissue from healthy controls [36–39, 41] and in NP tissue versus control tissue (ethmoid sinus or uncinate process) in those with CRSwNP [43, 52] (though there were exceptions [47, 48]). The increase in TSLP expression was consistent across geographical and ethnic groups, including patient populations from China, Italy, and the USA [36–39, 41, 43, 49, 52]. Most studies that compared NP tissue of patients with CRSwNP with uncinate tissue or unspecified nasal mucosal tissue from those with CRSsNP found increased TSLP mRNA expression in CRSwNP, with some tissue-specific differences in expression [36, 37, 39, 44]. Single-cell RNA sequencing of surgical CRS samples [58] and epithelial cell cultures [59] identified high TSLP expression levels in basal cells, and sequencing of basal cells obtained from patients with CRSwNP found higher TSLP expression than in cells of those with CRSsNP and greater numbers of TSLP+ basal cells [46]. TSLP receptor (TSLPR; *CRLF2*) mRNA expression was also found to be increased in the NP tissue of patients with CRSwNP compared with nasal mucosa tissue from healthy controls [36, 37].

Among patients with CRSwNP, TSLP mRNA expression has been shown to be increased in NP tissue and NECs isolated from those with eCRSwNP versus those with non-eosinophilic CRSwNP (neCRSwNP), which correlated with clinical severity of eCRSwNP [51, 53, 56, 57]. A Chinese study of ethmoid sinus tissue found that levels of TSLP and TSLPR mRNA were increased in patients with eCRSwNP compared with those with neCRSwNP and were also expressed more strongly in patients with CRS who were atopic (skin-prick test positive) than in those who were skin-prick test negative [57].

Overall, the literature indicates that TSLP mRNA expression is higher in patients with CRSwNP than in both those with CRSsNP and healthy controls, and higher in patients with eCRSwNP than in those with neCRSwNP. Additionally, this increased expression is localized to NPs and does not occur in adjacent tissue within the same patient.

### TSLP protein

Most studies have found increased levels of TSLP protein expression and/or secretion in the nasal epithelium of patients with CRSwNP versus those with CRSsNP and in patients with eCRSwNP versus those with neCRSwNP. Levels of TSLP were found to be increased in NP tissue and NECs from patients with CRSwNP compared with healthy controls [42, 45, 53]. Higher levels of TSLP were found in the nasal epithelial tissue of patients with CRSwNP than those with CRSsNP [44]. TSLPR was also found to be highly expressed in inflammatory infiltrate (composed of eosinophils, mast cells, and histiocytes) and epithelial cells in patients with CRSwNP and CRSsNP compared with tissue from healthy controls, with no significant difference between patients with CRSwNP and those with CRSsNP [40]. However, regarding epithelial cell expression of TSLPR, it should be noted that there is a lack of evidence that these cells respond to TSLP.

Multiple studies have shown that levels of TSLP and its receptor are higher in NP tissue and NECs from patients with eCRSwNP than those with neCRSwNP and healthy controls. TSLP levels were also higher in patients with neCRSwNP than in healthy controls [53–56, 60]. Expression of TSLP, TSLPR, and ST2L (suppression of tumorigenicity 2, the membrane-bound receptor for IL-33) correlated with symptom and CT scan scores in eCRSwNP. Additionally, TSLP, TSLPR, and ST2L expression correlated with the expression of Th2 cytokines in the nasal mucosa, suggesting a positive feedback loop between TSLP, IL-33, their cognate receptors, and Th2 cytokines that may drive Th2 inflammation in eCRSwNP [53]. In patients with eCRSwNP and neCRSwNP, protein expression of TSLP and both of its heterodimeric receptor subunits (TSLPR and IL-7Rα) was identified in eosinophils, plasma cells, and fibroblasts, as well as in NECs [57]. One study found that TSLP protein expression was associated with the degree of eosinophilic infiltration in the nasal gland duct epithelium but not the nasal mucosal epithelium [54].

Strong expression of TSLP, IL-33, and IL-25 proteins was observed in the nasal epithelium, small vessel endothelium, and subepithelial infiltrating cells of patients with severe asthma and CRS, whereas it was not found in those with non-severe asthma and CRS [49]. This corresponded with higher expression of T2 cytokines and greater numbers of ILC2s in patients with severe asthma and CRS [49]. Solitary chemosensory cells in NPs may be a key epithelial source of the IL-25 observed in NP tissue [61].

Observed increases in TSLP mRNA expression do not always correlate with increased levels of TSLP protein [36, 41]. Matched tissue proteomic and transcriptomic arrays demonstrated that several genes thought to be upregulated in NP versus uncinate tissue, including TSLP as well as IL-5, IL-13, CCL13, and CCL26, showed significant increases in mRNA but minimally or unchanged protein expression [41]. It was subsequently found that proteases found in NP tissue cleave TSLP into two products, which then dimerize to form a stable metabolite of TSLP with increased biological activity (in terms of mast cell, DC, and ILC2 activation) compared with full-length TSLP [36, 62]. Finally, one study analyzed TSLP levels in serum, finding no difference between patients with eCRSwNP and healthy controls [50].

Differences in sampling sites between studies are likely a primary driver of observed discrepancies in TSLP expression. Differing results could have also arisen from study differences in inclusion–exclusion criteria, preoperative treatment, geographical and ethnic composition, and microbiome composition. Furthermore, the generally low level at which both TSLP mRNA and protein are expressed presents a technical challenge for accurate detection and quantification across the different technology platforms utilized. Despite these limitations, the general consensus is that mRNA and protein expression of TSLP and TSLPR are increased in NP tissue of patients with CRSwNP versus those with CRSsNP and healthy controls and in patients with eCRSwNP versus those with neCRSwNP. Furthermore, TSLP expression has been found to correlate with eosinophilia and the severity of CRSwNP symptoms.

## Environmental and genetic influences on TSLP signaling in CRSwNP

CRSwNP is a condition of heterogeneous pathogenesis that, like severe asthma, is believed to arise from interactions between genetic predisposition and environmental factors. Genetic studies of TSLP in CRSwNP have been limited, though a single nucleotide polymorphism genotyping study in a Chinese cohort found that single nucleotide polymorphisms in the *TSLP* gene showed nominal significance with CRS risk, exerting a gender- and nasal polyposis-dependent association pattern [63]. Epigenetic mechanisms involving TSLP may also be relevant, with increased DNA methylation at the *TSLP* gene locus likely to be associated with CRSwNP pathogenesis [64]. Environmental factors, including exposure to inhaled pathogens, allergens, and irritants (such as viruses, bacteria, fungi, house dust mites, and cigarette smoke) have been shown to induce the expression of TSLP and other epithelial cytokines in the nasal epithelium of patients with CRSwNP.

### Viruses

Respiratory virus genomes are commonly found in secretions and tissue samples of patients with CRS [65]. NECs from sinus tissue of patients with CRSwNP release TSLP and IL-25 when stimulated with poly(I:C) (polyinosinic-polycytidylic acid), a synthetic analog of viral double-stranded RNA (dsRNA) [66]. Stimulation with poly(I:C) induced significantly higher expression of TSLP mRNA and protein in NECs from patients with NPs than in NECs from healthy controls, which may result from downregulation of the transcription factor dual specificity protein phosphatase 1 in NP NECs [67]. Similarly, dsRNA strongly induced the production of TSLP in NECs from patients with CRS, and IL-4 synergistically enhanced dsRNA-induced TSLP production [36]. Lastly, NECs from patients with CRS stimulated with viral or bacterial antigens exhibited increased production of TSLP and IL-6; conditioned media from the stimulated NECs enhanced activation of DCs and CD (cluster of differentiation) 4+ T cells and increased antigen-specific immunoglobulin (Ig)A and IgG production, at least in part through TSLP and IL-6 signaling [68].

### Bacteria

*S. aureus* exposure increased TSLP, IL-33, IL-5, and IL-13 expression in NP tissue via Toll-like receptor 2 signaling and increased the expression of TSLP and IL-33 receptors predominantly on CD3+ T cells [69]. In addition, *S. aureus* virulence factors, such as enterotoxin B, can also indirectly induce TSLP and IL-5 production in ethmoid explants from patients with CRSwNP (but not healthy controls) through increased expression of permeability glycoprotein, an efflux pump associated with cytokine transport [70].

### Allergens

Stimulation of healthy control NECs from inferior turbinate tissue with airborne allergens *Alternaria alternata*, *Aspergillus fumigatus*, *Dermatophagoides pteronyssinus,* and *D. farinae* (fungi and house dust mites) enhanced TSLP and IL-33 production via NF-κB (nuclear factor kappa-light-chain-enhancer of activated B cells), AP-1 (activator protein 1), and MAPK (mitogen-activated protein kinase) signaling. Despite increased production of TSLP and IL-33, NEC-conditioned media induced Th1 as well as Th2 cytokine production from ILC2s but did not have an impact on ILC2 numbers [55]. However, a study of NECs derived from NP tissue of patients with CRSwNP or ostiomeatal mucosa tissue of those with CRSsNP found no change in TSLP release upon stimulation with *A. fumigatus* and *D. pteronyssinus* [66]. There are several potential explanations for this finding, including higher baseline levels of TSLP in CRSwNP and CRSsNP tissue than in healthy control tissue, tissue-specific differences in expression, and technical factors [55]. Allergen-induced production of TSLP and IL-25 in NECs has been found to be mediated, at least in part, by the production of calprotectin; allergen-induced calprotectin production was increased in NECs from the NPs of patients with eCRSwNP versus neCRSwNP [71].

### Pollutants

Cigarette smoke has been shown to damage airway epithelial cells and increase TSLP levels in the bronchial epithelium and airway smooth muscle [72, 73]. Conditioned medium from human nasal epithelial cell cultures exposed to cigarette smoke extract significantly increased the secretion of TSLP in treated monocyte-derived DCs [74]. Similarly, particulate matter with an aerodynamic diameter of <2.5 μm (PM2.5) has been associated with airway epithelial cell injury, nasal epithelial barrier damage, and respiratory diseases, and has been shown to increase TSLP expression in cultured NECs from healthy controls [75]. However, in NECs isolated from patients with eCRSwNP, PM2.5 stimulation resulted in a dose-dependent decrease in TSLP mRNA and no associated change in TSLP protein, potentially owing to the higher baseline concentration of TSLP in cultures from patients with CRSwNP than in those from healthy controls [76] [. Hypoxia is now thought to contribute significantly to the pathogenesis of CRS via the stabilization and accumulation of intracellular hypoxia-induced factor 1a (HIF-1a) protein, which translocates to the nucleus where it can modulate the expression of target genes including *TSLP.* In human NECs, hypoxic conditions led to the upregulation of HIF-1a and the subsequent upregulation of TSLP, IL-25, and IL-33 mRNA and downstream T2 cytokines [60].

## Cytokines regulating TSLP expression in CRSwNP

The regulation of TSLP signaling in CRSwNP remains to be fully elucidated. However, current evidence suggests that pro-inflammatory cytokines like oncostatin M (OSM) and TNF-α work in synergy with the T2 cytokines IL-4 and IL-13 to promote increased TSLP production in NECs and fibroblasts from patients with CRSwNP versus those from healthy controls, further potentiating the inflammatory response [39, 77]. In NP tissue from patients with CRSwNP, TSLP production can be inhibited by immunosuppressive cytokines IL-37 and IL-10, as well as interferon-γ [77].

Basal stem cells co-expressing higher levels of TSLP and basal cell adhesion molecule were increased in the sinonasal mucosa of patients with CRSwNP versus those with CRSsNP, and their differentiation was prevented by a positive feedback loop in which basal cell adhesion molecule expression is reinforced by IL-4 and IL-13 [46].

Elevated endogenous protease inhibitor cystatin SN (CST1; a protein that protects against allergen, viral, and bacterial proteases) in NECs from patients with eCRSwNP versus those with neCRSwNP acted via a positive feedback loop with TSLP and IL-33 to amplify eosinophilic infiltration and T2 inflammation, and was associated with disease severity [78]. IL-37 secretion was inhibited in patients with eCRSwNP, resulting in TSLP production in NECs and increased eosinophilia [79].

Decreased cystatin A and serine peptidase inhibitor type 5 (SPINK5) levels in NECs from patients with eosinophilic CRS versus those with non-eosinophilic CRS or healthy controls promoted allergen-induced production of TSLP [80]. Inhibition of programmed cell death 4 expression by microRNA (miR)-21 decreased TSLP expression by inducing IL-10 production and inhibiting the activation of NF-κB in NP NECs [38].

## Downstream effects of TSLP signaling in CRSwNP

**Figure 2.**
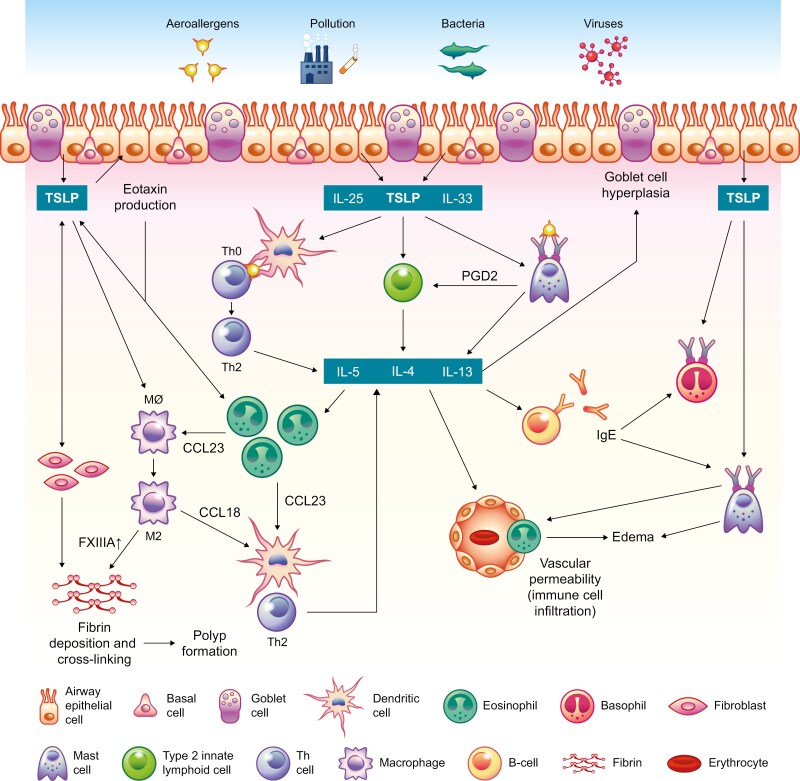
Mechanisms of TSLP signaling in T2/eosinophilic CRSwNP. In response to inhaled allergens and pathogens, nasal epithelial cells secrete TSLP, IL-25, and IL-33. TSLP stimulates the innate immune response via the promotion of T2 cytokine secretion by ILC2s and polarizes dendritic cells, driving the differentiation of naive T cells to Th2 cells that produce T2 cytokines. TSLP can also act directly on eosinophils, mast cells, basophils, fibroblasts, and macrophages to further drive T2 cytokine production. T2 cytokine signaling can lead to goblet cell hyperplasia and increased mucus production, edema, fibrin deposition and crosslinking, and subsequently nasal polyp formation. CCL, CC chemokine ligand; CRSwNP, chronic rhinosinusitis with nasal polyps; FXIIIA, clotting factor XIIIa; IgE, immunoglobulin E; IL, interleukin; M, macrophage; PGD2, prostaglandin D2; T2, type 2; Th, T helper; TSLP, thymic stromal lymphopoietin.

TSLP initiates and amplifies T2 inflammation in the airway and is also involved in airway remodeling processes. The pleiotropic effects of TSLP signaling on eosinophils, DCs, Th2 cells, ILC2 cells, mast cells, basophils, macrophages, and fibroblasts in patients with CRSwNP are outlined in **Figure 2**.

### Eosinophils

Activated eosinophils release extracellular traps, which have been shown to induce airway epithelial cell desquamation, increase barrier permeability, and increase the release of TSLP, contributing to a vicious cycle of inflammation [81, 82]. Eosinophils express TSLPR and IL-7Rα, with TSLP promoting eosinophil survival, activation, adhesion, and release of IL-6 through extracellular signal-regulated kinase, p38 MAPK, and NF-κB signaling pathways [83, 84]. TSLP can also act on eosinophils indirectly, via upregulation of IL-5 (which promotes eosinophil survival and activation) by mast cells, ILC2s, and Th2 cells [34, 85]. In line with this, treatment with anti-TSLP monoclonal antibodies reduces blood eosinophil counts and airway submucosal tissue eosinophil numbers in patients with asthma [86–88]. Eosinophilic inflammation in patients with CRSwNP (as well as those with asthma) is also regulated by T2 cytokines that induce epithelial cells to produce eotaxins, which recruit eosinophils to the airway via chemokine receptor 3 (CXCR3) binding [89]. Eotaxin levels are higher in patients with eCRSwNP than in those with neCRSwNP or healthy controls [90, 91]. TSLP stimulation of NECs from patients with eCRSwNP increased the expression of CCL11 (eotaxin-1) in NECs via the Janus kinase (JAK)1/2-signal transducer and activator of transcription (STAT)3 signaling pathway, which might contribute to eosinophilic infiltration in patients with eCRSwNP [89]. TSLP has also been found to be involved in endothelial microRNA-regulated eosinophil trafficking in patients with CRSwNP [92]. Serum miR-1 levels were inversely correlated with tissue eosinophilia in samples from patients with CRSwNP, and miR-1 recruited TSLP (and P-selectin, CCL26 [eotaxin-3], and thrombopoietin receptor) to the RNA-induced silencing complex and downregulated these genes [92]. Plasma CCL26 levels may be a useful biomarker for eCRSwNP because they are associated with NP tissue eosinophilia and plasma TSLP and IL-33 levels in patients with eCRSwNP [93].

Eosinophils also appear to play a role in the TSLP-mediated development of corticosteroid resistance in CRSwNP. Despite widespread use, corticosteroids may only be effective in 50%–80% of patients with CRSwNP, with some patients exhibiting resistance to treatment [94, 95]. NECs collected from patients with CRSwNP who were also corticosteroid resistant (defined as an NP reduction of 1 NP endoscopic score or less after 14 days of OCS treatment) expressed high levels of livin. This high expression of livin, in conjunction with Ras activation, induced TSLP expression in NECs [96]. TSLP exposure induced eosinophils and neutrophils to express glucocorticoid receptor β, which mediates competitive inhibition of steroid binding and leads to the development of corticosteroid resistance [96].

### DCs, Th2 cells, and Th2 progenitors

DCs are antigen-presenting cells that play an important role in linking innate and adaptive immune responses [97]. TSLP-activated DCs have been shown to preferentially activate Th2 cells in an OX40L-dependent manner [98], stimulating them to produce inflammatory cytokines, including IL-4 and TNF [34]. DCs isolated from the nasal mucosa of patients with CRSwNP and primed with TSLP induced the differentiation of naive CD4+ T cells into TNF+IL-4+ T cells. Conversely, DCs co-primed with TSLP and lipopolysaccharide promoted the development of TNF+IFN-γ+ T-cells [44]. Higher expression of TSLPR and its downstream effector OX40L was detected in DCs isolated from the nasal mucosa of patients with NP than from those without NP [44]. OX40L mRNA and protein expression were also increased in NPs of patients with CRSwNP versus nasal mucosa from those with CRSsNP; moreover, this increase was correlated with eosinophilia [37].

Distinct subsets of lesional DCs have been shown to exist in eCRSwNP and neCRSwNP, with different Th-cell polarizing functions. In eCRSwNP, OX40L/programmed death ligand 1-positive DCs primed Th2 cells, whereas in neCRSwNP, OX40L/programmed death ligand 1-low-expressing DCs primarily induced Th1/Th17 cells [51]. TSLP mRNA expression was upregulated in patients with eCRSwNP versus those with neCRSwNP or healthy controls, and this upregulation was correlated with the percentage of OX40L+ DCs in the sinonasal mucosa [51].

Th2 multipotent progenitor (Th2-MPP) cells are a population of aberrant Th2 cells with features of chronic activation. Whereas Th2-MPP cells isolated from NP persisted even in the presence of IL-4 receptor blockade, TSLP drove their selective expansion and rendered them insensitive to corticosteroid-induced apoptosis *in vitro* [99]. Corticosteroid treatment also upregulated TSLPR expression in Th2-MPP cell cultures, suggesting that TSLP may act in a feedback loop to promote a steroid-resistant, inflammatory disease state [99].

### ILC2 cells

ILC2 cells can produce T2 cytokines via innate immune stimulation and are therefore considered to be a key orchestrator of airway T2 inflammation [100]. ILC2s have been shown to be elevated and activated in NPs, releasing T2 cytokines [85]. In CRSwNP, TSLP and IL-33 activate ILC2s, driving the release of T2 cytokines, such as IL-4, IL-5, and IL-13, via activation of NF-κB, STAT5, and GATA binding protein 3 signaling [85, 101, 102].

### Mast cells

Activated mast cells in the nasal epithelium release mediators (including T2 cytokines, cysteinyl leukotrienes, and histamine) that promote inflammation, vascular permeability, and local edema, which can obstruct nasal airways and lead to congestion in CRS [103]. The role of mast cells in CRSwNP and any associated role of TSLP have not been well studied; however, mast cells can both produce and respond to TSLP [104]. Subepithelial mast cells have been shown to proliferate in CRSwNP and acquire distinct phenotypes during T2 inflammation [103, 105]. Moreover, in the lamina propria of Chinese patients with eCRSwNP, most TSLP+ cells were found to be mast cells, and most TSLPR+ cells were found to be mast cells, DCs, macrophages, and CRTH2+ cells [53]. TSLP treated with NP extracts from patients with CRSwNP induced greater IL-5 production in mast cells than non-treated TSLP [36]. Furthermore, NP extracts significantly enhanced IL-1β-dependent IL-5 production in mast cells compared with uncinate tissue homogenates. These responses were significantly inhibited by anti-TSLP, suggesting that NPs contain biologically relevant levels of TSLP activity [36].

In patients with AERD, TSLP mRNA expression in NP tissue correlated strongly with markers of mast cells and prostaglandin D_2_ (PGD2), a major inflammatory mediator produced by mast cells [106]. Levels of the cleaved active form of TSLP protein were increased in NPs of patients with AERD relative to those from aspirin-tolerant controls. Moreover, TSLP induced PGD2 generation by cultured human mast cells. Overall, these findings indicate that mast cell-derived PGD2 is a major effector of T2 immune responses driven by TSLP and that dysregulation of this system contributes to AERD [106].

### Basophils

Although basophils are known to play an important role in T2 inflammation in CRSwNP [1, 107], our literature search did not identify any data linking TSLP directly with basophils in this condition. This gap may be a valuable avenue for future research. In contrast, evidence from other inflammatory conditions, such as eosinophilic esophagitis, suggests a role for TSLP in basophil activation and initiation of T2 pathways, which are known to drive pathophysiological alterations to the epithelium [108].

### Macrophages

TSLP can induce M2 macrophage polarization indirectly through IL-4 and IL-13 with downstream effects that include Th2 cell activation and tissue remodeling [109, 110]. Although our search yielded limited literature directly linking TSLP with macrophages in CRSwNP, macrophages in the lamina propria of Chinese patients with eCRSwNP have been observed to express TSLPR [53].

### Airway remodeling

Although our literature search did not identify any data regarding TSLP’s role in remodeling in CRSwNP specifically, TSLP is known to influence airway remodeling in asthma [35, 111] and in NEC cultures from patients with AR [112]. In the asthmatic airway, TSLP has been shown to activate lung fibroblasts, which produce extracellular matrix proteins, such as fibrin and matrix metalloproteases, that contribute to the thickening and fibrosis of the subepithelial basement membrane [113–115]. TSLP can also induce EMT in epithelial cells via upregulation of transforming growth factor β, causing them to lose their epithelial identity and migrate to the lamina propria where they acquire a fibroblast-like mesenchymal fate. These fibroblast-like cells then further contribute to tissue remodeling [112, 116]. EMT can also disrupt intracellular adhesions, impairing the epithelial barrier [112, 116, 117]. Epithelial barrier disruption allows pathogens to infiltrate the epithelium, triggering fibrogenic cytokine release that further decreases the expression of junctional proteins and drives a cycle of epithelial damage, airway remodeling, and chronic inflammation [118, 119]. By promoting IL-13 production, TSLP could play a similar role in driving EMT in CRSwNP.

Studies have shown that TSLP can induce the proliferation of vascular endothelial cells [120, 121] and promote the release of vascular endothelial growth factor-A from lung macrophages [122]. Inflammatory angiogenesis can lead to airway wall edema and NP development [123]. IL-4 and IL-13 signaling downstream of TSLP can result in goblet cell hyperplasia and increased mucus production, as well as impaired ciliary clearance of mucus [124–128]. Further research on the role of TSLP in airway remodeling in CRSwNP is required.

## TSLP as a therapeutic target in CRSwNP

Recommended treatments for CRSwNP include long-term intranasal corticosteroids and short-term oral corticosteroids, alongside saline solution rinses [129]. In certain cases, oral antibiotics and leukotriene receptor antagonists may also be prescribed [129]. Corticosteroids exert a therapeutic effect in CRSwNP by inhibiting inflammatory mediators, modulating T cells’ immune responses, particularly those involving Th2 cells and T2 cytokines, and reducing eosinophil infiltration [130]. These processes are known to be initiated and augmented by TSLP signaling [9, 32–34]. Indeed, several therapeutics for CRSwNP, such as clarithromycin, dexamethasone, and montelukast, have demonstrated effects on TSLP signaling [131–134].

In recent years, the growth of biological therapies targeting factors associated with T2 inflammation has expanded the number of potential treatments for patients with CRSwNP [135, 136]. Notably, three monoclonal antibodies targeting downstream inflammatory pathways or cellular mediators—omalizumab (anti-IgE), mepolizumab (anti-IL-5), and dupilumab (anti-IL-4Ra)—are approved by the US Food and Drug Administration for use in these patients [137, 138].

TSLP, which sits at the top of the inflammatory cascade and stimulates the release of T2 mediators targeted by the approved biologics, is a promising target in CRSwNP treatment. The WAYPOINT phase 3, randomized, placebo-controlled trial evaluated the efficacy and safety of the anti-TSLP monoclonal antibody tezepelumab in patients with severe CRSwNP, with or without comorbid asthma (NCT04851964). WAYPOINT met both primary endpoints, with tezepelumab recipients exhibiting significant improvements in the total NP score (mean difference versus placebo, −2.07; 95% confidence interval [CI], −2.39 to −1.74) and the mean nasal-congestion score (−1.03; 95% CI, −1.20 to −0.86) after 52 weeks’ treatment (*P* < 0.001 for both scores) [139]. Tezepelumab also improved the loss-of-smell score, Sino-Nasal Outcome Test-22 total score, Lund-Mackay score, and total symptom score versus placebo. Furthermore, treatment with tezepelumab resulted in a near-complete elimination of the need for treatment intervention with SCS and/or surgery, and there were no new safety concerns [139].

Further relevant data regarding the impact of TSLP blockade in CRSwNP come from the phase 3 NAVIGATOR study of tezepelumab in patients with severe uncontrolled asthma. NAVIGATOR participants with a history of NPs in the 2 years before randomization had reduced levels of IgE, IL-5, and IL-13, and improved Sino-Nasal Outcome Test-22 scores after 52 weeks of treatment (LS mean change from baseline of −21.06 points [standard error (SE): 2.50] for tezepelumab and −10.48 points [SE: 2.56] for placebo; LS mean difference with tezepelumab versus placebo, −10.6 [95% CI: −17.8, −3.4]) [140]. Similar results were observed for patients with severe uncontrolled asthma and any history of NPs (LS mean difference with tezepelumab versus placebo, −11.1 [95% CI: −17.8, −4.4]) [141]. Tezepelumab recipients also had an improved sense of smell, reduced nasal blockage and cough, increased productivity, and reduced incidences of waking up tired [142]. Additionally, in recipients of tezepelumab, asthma exacerbations were reduced by 85% (95% CI: 72%–92%) and lung function was improved versus placebo recipients [140].

Given the prominent role of TSLP in stimulating innate and adaptive immunity, targeting TSLP brings a theoretically increased risk of systemic immunosuppression and serious infections. However, in phase 2b/3 trials across multiple diseases (asthma, chronic obstructive pulmonary disease, CRSwNP, atopic dermatitis, chronic spontaneous urticaria), no increase in serious infections was observed with tezepelumab [88, 139, 143–146]. There was also no increase in anti-drug antibodies observed [88, 139, 143–146]. Furthermore, tezepelumab has been shown to reduce the airway epithelial inflammatory response to viral challenge without affecting anti-viral host resistance [147]. Observational studies and post-marketing surveillance currently in progress will provide additional safety data in a real-world context.

## Conclusion and future perspectives

This review has considered current evidence for the role of TSLP in the pathophysiology of CRSwNP, finding that TSLP and TSLPR are overexpressed in various cell types within the sinonasal mucosa of patients with this condition. Furthermore, TSLP signaling initiates and amplifies the chronic T2-skewed inflammation that drives sinonasal symptoms. Anti-TSLP monoclonal antibody therapy with tezepelumab has demonstrated high levels of efficacy, as well as safety, in patients with severe CRSwNP. By targeting TSLP at the top of the immunological cascade, tezepelumab appears to comprehensively address multiple pathways underlying CRSwNP disease biology. Further studies are ongoing to understand whether the improvements in disease activity with tezepelumab translate into slowing or halting disease progression, potentially restoring epithelial health and providing sustained on-treatment remission for patients with CRSwNP. Furthermore, the growing body of real-world evidence for biologic therapy in CRSwNP will provide valuable information on the long-term effectiveness and safety of these treatments. Given the heterogeneity of CRSwNP, improvements in our understanding of its pathogenesis could help characterize specific patient endotypes and identify noninvasive biomarkers, further assisting the targeting of precision therapies such as biologics and transforming the therapeutic landscape of this chronic condition.

## Data Availability

No new data were generated or analyzed in support of this research.
